# The role of internet-based therapies in adolescents’ quality of life: a systematic review and meta-analysis

**DOI:** 10.1186/s40359-026-04285-z

**Published:** 2026-03-14

**Authors:** Yu Liu, Yang Yang, Qingxi Xu

**Affiliations:** 1 School of Intelligent Manufacturing, Nanyang Institute of Technology, Nanyang, Henan 473004 China; 2 School of Marxism, Nanyang Institute of Technology, Nanyang, Henan 473004 China

**Keywords:** Adolescents, Meta-analyses, Quality of life, Internet-based intervention

## Abstract

**Background:**

This meta-analysis assessed the impact of internet-based interventions on adolescents’ quality of life (QoL).

**Methods:**

A systematic electronic database searching was conducted through November 12, 2024, to identify pertinent studies. Randomized controlled trials evaluating internet-based interventions including cognitive-behavioral therapy, mindfulness training, and online peer support in adolescents aged 10–19 years were included. Studies were required to measure QoL using validated instruments with CHU9D, PedsQL, Brunnsviken Brief Quality of Life scale, or Health-Related Quality of Life scales and compare interventions against standard care, face-to-face therapy, or no treatment. Studies with participants outside the adolescent age range or without QoL outcomes were excluded. Effect sizes were quantified using standardized mean difference and standard error. Five studies were included, with 156 participants in the intervention group and 167 in the usual care group.

**Results:**

Five studies involving 323 participants were included. Overall, internet-based interventions showed a small and non-significant effect on adolescents’ quality of life. Subgroup analyses suggested that shorter interventions may be associated with modest improvements, whereas longer interventions showed inconsistent or potentially unfavorable effects. However, substantial heterogeneity was observed across studies, reflecting differences in populations, intervention types, and outcome measures.

**Conclusion:**

Internet-based interventions do not demonstrate a consistent overall benefit for adolescents’ quality of life. Given the considerable heterogeneity and limited number of studies, findings should be interpreted with caution. In clinical practice, careful consideration of intervention duration and developmental stage is recommended when implementing internet-based programs for adolescents.

**Supplementary Information:**

The online version contains supplementary material available at 10.1186/s40359-026-04285-z.

## Introduction

The internet is now an integral part of daily adolescent life, shaping social relationships, access to information, and personal development [1]. Often referred to as digital natives, adolescents navigate a highly networked environment in which digital media influence multiple aspects of everyday functioning [2]. While the internet facilitates social connection, learning opportunities, and skill development, it also poses potential risks to adolescents’ physical, social, and emotional well-being [3]. Consequently, understanding how digital technologies may be harnessed to improve adolescents’ quality of life (QoL) has become an important public health concern [4].

Adolescence represents a critical developmental period marked by profound physical, emotional, and social changes as individuals transition from childhood to adulthood [5]. During this stage, adolescents are exposed to multiple stressors, including academic demands, identity formation, and evolving interpersonal relationships, all of which influence overall QoL [6]. QoL is a multidimensional construct encompassing physical health, psychological well-being, social functioning, and life satisfaction [7]. Factors such as mental and physical health status, peer and family relationships, and academic experiences collectively shape adolescents’ perceived QoL [8]. Mental health concerns are particularly salient during adolescence, as this period often coincides with the first onset of psychiatric symptoms [9]. A substantial proportion of adolescents experience mental disorders, including anxiety disorders, eating disorders, and depression [10]. These conditions can negatively affect emotional functioning, academic performance, social relationships, and self-esteem, thereby substantially diminishing QoL [9, 11, 12]. Importantly, adolescence is also the developmental window during which serious mental illnesses, such as schizophrenia and bipolar disorder, commonly begin to emerge. These conditions may present unique challenges for intervention, and it remains unclear whether internet-based treatments are appropriate or effective for adolescents with such severe or complex psychopathology. This limitation highlights an important boundary within the current evidence base.

In addition to mental health challenges, physical health conditions significantly influence adolescents’ QoL. Chronic diseases such as obesity, diabetes, and asthma have become increasingly prevalent in this population [13]. These conditions can restrict physical activity, require ongoing medical management, and negatively affect psychosocial well-being [14, 15]. Given the multifaceted nature of adolescent well-being, interventions must address both psychological and physical dimensions of health [16].

The study defines Internet-based interventions (IBIs) as structured programs which deliver psychological and behavioral treatment through secure websites and web portals and digital applications [17]. The interventions can include cognitive-behavioral therapy and acceptance and commitment therapy and mindfulness-based approaches and psychoeducation and structured self-management programs which can be delivered through self-guidance or therapy assistance [18–20]. To meet inclusion criteria, interventions were required to be delivered predominantly online, structured into modules or sessions, and compared with standard care, face-to-face therapy, or no-treatment control conditions. Purely face-to-face interventions or programs without a structured therapeutic framework were excluded. Although researchers have conducted numerous studies about IBI effectiveness in treating adolescents, they have mainly investigated how the treatment affects specific symptoms of different disorders While symptom improvement is clinically important, it does not fully capture broader functioning and well-being. QoL represents a multidimensional construct encompassing physical, psychological, and social functioning, as well as overall life satisfaction. Because QoL reflects adolescents’ global adaptation and daily functioning, it provides a more comprehensive and patient-centered outcome than symptom measures alone [21, 22].

Despite increasing implementation of internet-based programs, relatively few studies have synthesized their effects on QoL as a primary outcome [23]. Existing trials often report QoL as a secondary endpoint or focus narrowly on specific clinical symptoms, leading to fragmented evidence. The combination of small study samples and diverse research methods used in individual studies restricts this study from making definitive conclusions. A meta-analysis is needed to quantitatively combine existing research data which will determine how IBIs affect adolescents’ quality of life and identify factors that cause different results in research studies.

## Methods

### Search strategy

Databases including PubMed, Scopus, and Web of Science (WOS) were systematically searched to identify relevant studies. The search strategy combined terms related to “quality of life”, AND “internet-based intervention” OR “web-based intervention” OR “online intervention”, AND “adolescent” OR “youth”, OR “teen”. Boolean operators (AND, OR) were applied as appropriate across databases. No review protocol was prospectively registered in PROSPERO; however, the search strategy, eligibility criteria, and analytical approach were defined a priori and are fully reported to ensure methodological transparency. Titles and abstracts of retrieved records were screened by one author to assess eligibility, and studies meeting the inclusion criteria were subsequently evaluated through full-text review.

### Study selection

Duplicate records were first removed using EndNote. The remaining studies were independently by three researchers against the pre-specified eligibility criteria, using a systematic and strict selection process. The intervention was IBIs, such as CBT, mindfulness training, and online peer support. The comparison was between control groups receiving standard care, face-to-face therapy, or no treatment. The outcome measured QoL change using standardized psychological assessments. Lastly, the Study Design was randomized controlled trials (RCTs) and controlled clinical trials. Studies needed to have a clearly defined research objective, report relevant findings, and contain data regarding the intervention’s impact on adolescent QoL. Publications were restricted to the English language, and the search included articles through November 12, 2024. The inclusion and exclusion criteria are summarized in Table 1. Three reviewers independently reviewed titles and abstracts to select included studies. Disagreements were resolved through discussion and consensus. The third researcher was available to settle the last of the discrepancies, but was ultimately not needed. This procedure ensured that only studies that met the inclusion criteria were selected, maximizing the validity and reliability of the analysis.

**Table 1:** Inclusion and exclusion criteria summary

Domain	Inclusion Criteria	Exclusion Criteria
Population	Adolescents aged 10–19 years	Participants younger than 10 or older than 19 years
Intervention	Internet-based interventions	Face-to-face interventions only or non-IBIs
Comparison	Standard care, face-to-face therapy, or no treatment	Unspecified or inappropriate comparison group
Outcome	Quality of Life measured using validated instruments	Studies without QoL outcomes
Study Design	RCTs and controlled clinical trials	Observational, qualitative, case reports, or non-controlled trials

### Data extraction

Three reviewers searched the literature systematically against predetermined inclusion criteria. Although formal kappa statistics were not calculated, two reviewers independently screened the identified studies before comparing the findings. Disagreement about including studies was resolved by discussion and consensus, and a third reviewer would serve as a mediator of unresolved conflicts. Once the selection process had been completed, the reviewers proceeded to data extraction, where they followed a systematic and structured process. For each study, study characteristics, participant characteristics, intervention details, and outcome measures including mean and SD for intervention and control groups at baseline and post-intervention were extracted. Change scores were calculated where applicable. In arranging the data retrieved in an organized pattern, this method made it accurate and consistent, allowing for a strong and sound analysis of studies in the review.

### Quality assessment

The Cochrane Collaboration’s Risk of Bias Assessment Tool (RoB 2) [24], which covered the following areas, was utilized to assess the quality of included studies comprising (D1) bias related to the randomization procedure; (D2) bias due to deviation from the planned interventions; (D3) bias due to missing data on outcomes; (D4) bias in measurement of outcome; and (D5) bias due to reporting selection. Within each domain and as a whole, included studies were classified as having low risk, some concerns, or high risk of bias. The risk of bias of included studies was assessed independently by two reviewers using the Cochrane Risk of Bias 2 (RoB 2) tool. Each reviewer evaluated all domains separately. Any discrepancies in judgments were discussed and resolved through consensus. If disagreement persisted, third reviewer was consulted to reach a final decision. This procedure ensured independent assessment and minimized subjective bias in the evaluation process. Further details about Risk of bias can be found in supplementary material (Supplementary Table S2).

### Statistical analysis

All statistical analyses were conducted using IBM SPSS Statistics (version 31). Data were combined based on a random-effects model, which accommodates research heterogeneity. The primary effect size for continuous outcomes was the standardized mean difference (SMD), where appropriate, derived from raw data. Effect sizes were reported with 95% confidence intervals (CI), and the inverse-variance approach was used for weighting. Effect sizes were modified as needed to take into consideration group interdependence utilizing a predetermined correlation-based methodology. Heterogeneity was evaluated using ${\mathrm{H}}^{2}$, ${\mathrm{I}}^{2}$, and $\mathrm{T}\mathrm{a}{\mathrm{u}}^{2}$. Tau² calculates the between-study variance, I² shows the proportion of variation across trials attributable to heterogeneity rather than chance (values ≥ 50% indicating considerable heterogeneity), and H² quantifies the excess variability in effect sizes beyond sampling error. Studies were categorized according to duration during the subgrouping step. To address variability and identify patterns and differences that might not be seen in a pooled analysis, this step was crucial. The relationship between effect size and research variables was visualized using a bubble plot, which especially looked at how sample size and methodological quality affected results. A forest plot showed the combined SMD and its associated CI. Funnel plot was used to visually assess potential publication bias by examining the symmetry of study effect sizes around the overall pooled estimate; marked asymmetry may suggest the presence of publication bias, whereas symmetry indicates a lower likelihood of such bias.

## Results

### Study selection

**Fig. 1:**
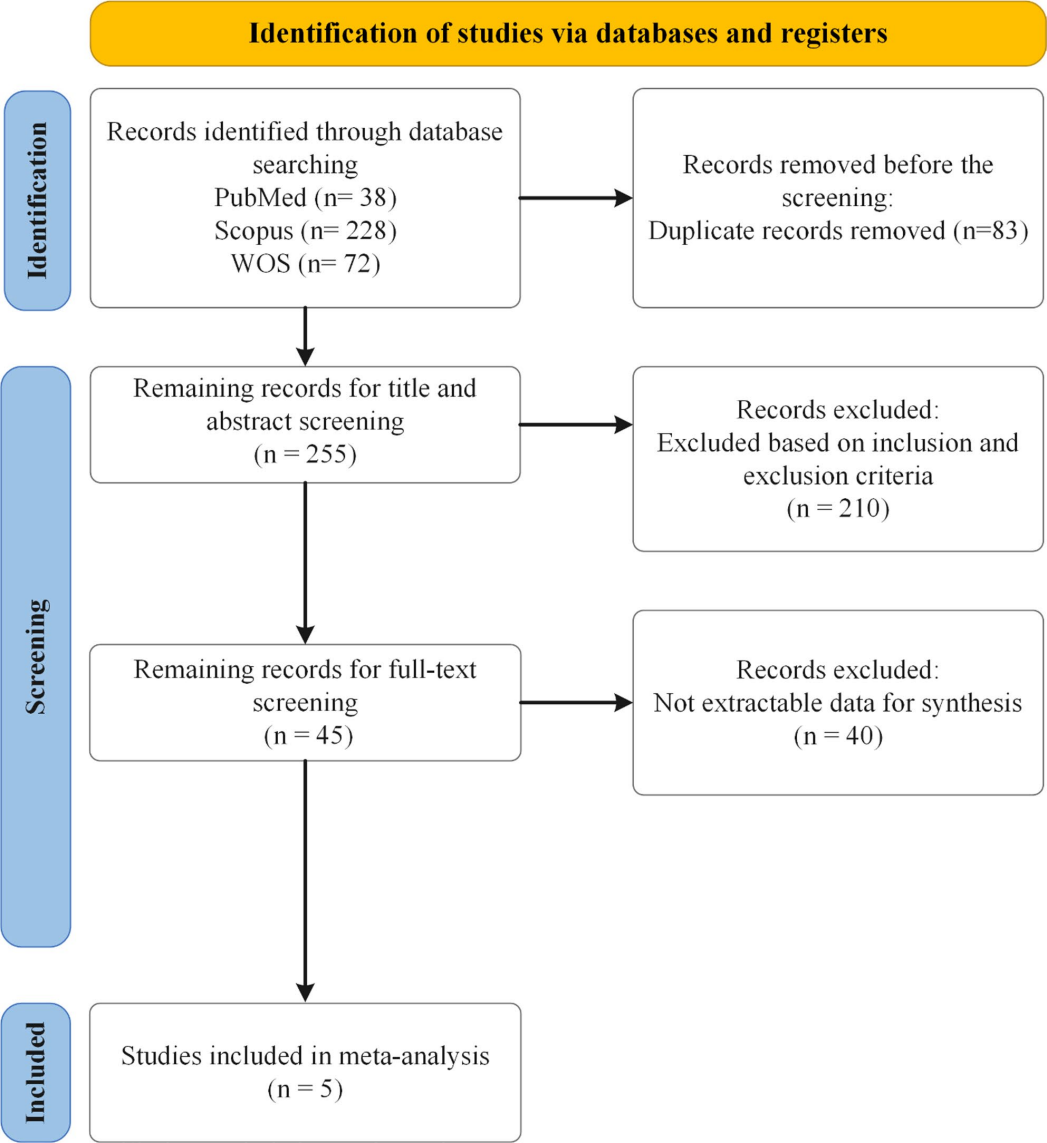
The included studies flowchart

A comprehensive literature search was conducted across three major electronic databases including PubMed, Scopus, and WOS mentioned in Fig. 1. The primary aim of this search was to identify relevant studies examining internet-based interventions in adolescents and their impact on QoL. A systematic approach, following the Preferred Reporting Items for Systematic Reviews and Meta-Analyses guidelines (PRISMA) [25], was used to ensure the inclusion of high-quality and relevant studies for meta-analysis. During the identification stage, a total of 338 records were retrieved from the databases: 38 from PubMed, 228 from Scopus, and 72 from WOS. To remove redundancies, 83 duplicate records were removed before proceeding to the screening phase. In this phase, the remaining 255 records were reviewed based on their titles and abstracts. After an initial assessment, 210 studies were excluded due to a lack of relevance to the research topic. During the eligibility assessment, 45 full-text articles were examined. At this stage, 40 articles were excluded, 7 due to lack of access and 33 because they did not measure QoL. Ultimately, 5 studies met al.l inclusion criteria and were incorporated into the final quantitative synthesis. This structured search strategy, guided by PRISMA, ensured a rigorous selection process, enhancing the reliability and validity of the study findings. Further information about PRISMA chart and check list can be found in supplementary material (Supplementary Fig. S1 and Table S1).

### Characteristics investigations

A total of five studies met the inclusion criteria and were selected for further analysis, as summarized in Table 2 [26–30]. These studies were published between 2016 and 2023 and were conducted across four countries comprising Sweden, Canada, Spain, and Germany. The included investigations were implemented within diverse clinical and healthcare contexts, including specialist outpatient mental health clinics, pediatric subspecialty settings, primary care–linked clinical populations, and nationwide home-based internet-delivered programs.

**Table 2:** A summary of the included research

Author	Year	Country	Study context / setting	IG sample size	CG sample size	Type of Therapy	Intervention Components	Delivery Format	Guidance/Support	Duration	Mean Age	Instruments and outcome
[26]	2021	Sweden	Specialist outpatient mental health clinic with internet-delivered intervention	51	52	Internet-delivered cognitive behavioral therapy	10-module CBT for social anxiety + 5 parent modules. Psychoeducation, exposure, social skills, attention shifting, cognitive restructuring, relapse prevention.	Secure web platform. Asynchronous modules. Three video calls. Secure messaging.	Minimal therapist support. Three video calls. Asynchronous messaging throughout. Excellent fidelity.	22 Weeks	14.1	Child Health Utility 9D
[27]	2016	Canada	Specialist outpatient pediatric rheumatology setting with home-based internet intervention	22	24	Internet-based	12-module self-management program including arthritis education, symptom management, relaxation, stress management, lifestyle. Goal-setting, quizzes, homework.	Web-based, password-protected. Weekly modules. Interactive content, video/audio, email, discussion board.	Weekly telephone calls by trained coach. Email support. Daily discussion board monitoring. Reviewed homework and goals.	12 Weeks	14.6	Pediatric Quality of Life Inventory
[28]	2020	Spain	Clinical pediatric population with home-based web-based psychosocial intervention	25	36	Web-Based Psychosocial Intervention	7-unit CBT program for child-parent dyads with pain education, goal-setting, relaxation, communication, cognitive restructuring. Multimedia with comics.	IRIS web platform. Self-directed, asynchronous. Multimedia, personalized content.	Self-directed. Reminders only if inactive > 10 days. Technical support available. No active therapist feedback or calls.	11 Weeks	11.11	Pediatric Quality of Life Inventory
[29]	2023	Sweden	Primary care–linked clinical population with nationwide home-based internet intervention	27	25	Internet-delivered acceptance and commitment therapy	8-module ACT/CBT program with psychoeducation, values, exposure, defusion, mindfulness. Text, video/audio, exercises, homework.	Web platform. Asynchronous, self-paced. Text, video/audio, interactive exercises.	Guided by supervised students. Weekly written feedback, 3 phone calls. Motivational contact if inactive.	10 Weeks	16.63	Brunnsviken Brief Quality of Life Inventory
[30]	2021	Germany	Specialist outpatient pediatric cardiology population with home-based eHealth exercise intervention	31	30	E-Health Exercise Intervention	Video exercise sessions with automated tracking.	Asynchronous online platform. Video-based, self-paced.	Self-guided. Weekly emails. Phone contact if inactive > 2 weeks.	24 Weeks	13	Health-related Quality of Life

Sample sizes in the intervention groups ranged from 22 to 51 participants, while control group sizes ranged from 24 to 52 participants. Intervention durations varied substantially, ranging from 10 to 24 weeks. As detailed in Table 2, the interventions varied considerably in their components, delivery formats, and levels of therapist support. The interventions comprised internet-delivered cognitive behavioral therapy, acceptance and commitment therapy, web-based psychosocial interventions, internet-based self-management programs, and eHealth exercise programs. Intervention components ranged from 7 to 12 modules covering diverse therapeutic elements including psychoeducation, exposure therapy, cognitive restructuring, relaxation training, social skills development, mindfulness, and behavioral activation. Delivery formats included secure web platforms with varying media types, with most interventions delivered asynchronously and self-paced. Therapist support varied from minimal or absent to more intensive guidance involving weekly written feedback and scheduled telephone or video calls.

QoL outcomes were assessed using validated instruments appropriate for adolescent populations. These included the Child Health Utility 9D (CHU9D), the Pediatric Quality of Life Inventory (PedsQL), the Brunnsviken Brief Quality of Life Inventory (BBQ), and standardized health-related quality of life (HRQoL) outcome measures. These instruments assessed multiple dimensions of QoL, including physical, emotional, and psychosocial functioning. Participants were adolescents, with mean ages ranging from 11.11 to 16.63 years. The variation in clinical populations, intervention settings, delivery modalities, therapist support models, and national healthcare contexts highlights the substantial heterogeneity of the included studies and underscores the importance of contextual considerations when interpreting the effects of internet-based interventions on adolescent QoL. Detailed intervention characteristics for each study are presented in Table 2, which provides comprehensive information about intervention components, delivery formats, and guidance mechanisms to facilitate interpretation of the meta-analytic findings.

### Quality of research techniques

**Fig. 2:**
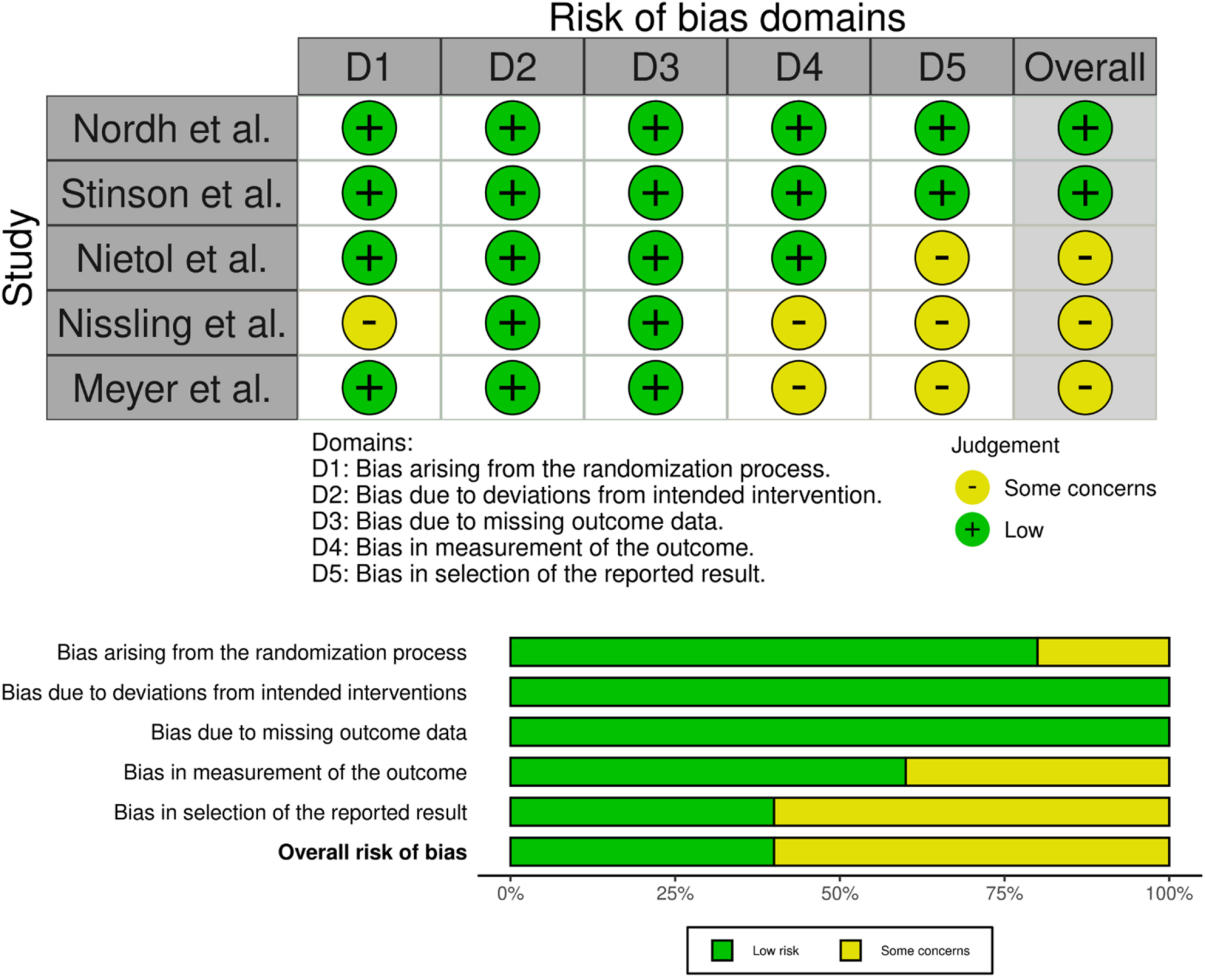
Risk of bias assessment for included studies

The risk of bias of the included studies was assessed across five domains using the RoB 2 framework, as illustrated in Fig. 2. These domains covered the randomization process (D1), deviations from intended interventions (D2), missing outcome data (D3), measurement of outcomes (D4), and selection of reported results (D5). Overall, 40% of the studies were judged to be at low risk of bias, while 60% showed some concerns. Importantly, no study was classified as having a high risk of bias in any domain, indicating generally acceptable methodological quality. Regarding the randomization process (D1), 80% of studies were rated as low risk, with the remaining 20% showing some concerns, mainly due to limited reporting of allocation procedures rather than clear methodological flaws. All studies (100%) were rated as low risk for deviations from intended interventions (D2) and missing outcome data (D3), suggesting that interventions were implemented as planned and that outcome data were handled appropriately. For measurement of outcomes (D4), 60% of studies were at low risk, while 40% raised some concerns, primarily related to insufficient detail about blinding of outcome assessors or measurement procedures. The selection of reported results (D5) showed the greatest uncertainty, with only 40% of studies rated as low risk and 60% presenting some concerns, reflecting incomplete reporting of analysis plans or selective outcome reporting. While most studies demonstrated strong methodological rigor in intervention delivery and data completeness, some concerns remained regarding outcome measurement and selective reporting. These issues should be considered when interpreting the findings, although the absence of high-risk judgments supports the overall credibility of the evidence base.

### Meta-analyses of study results

**Fig. 3:**
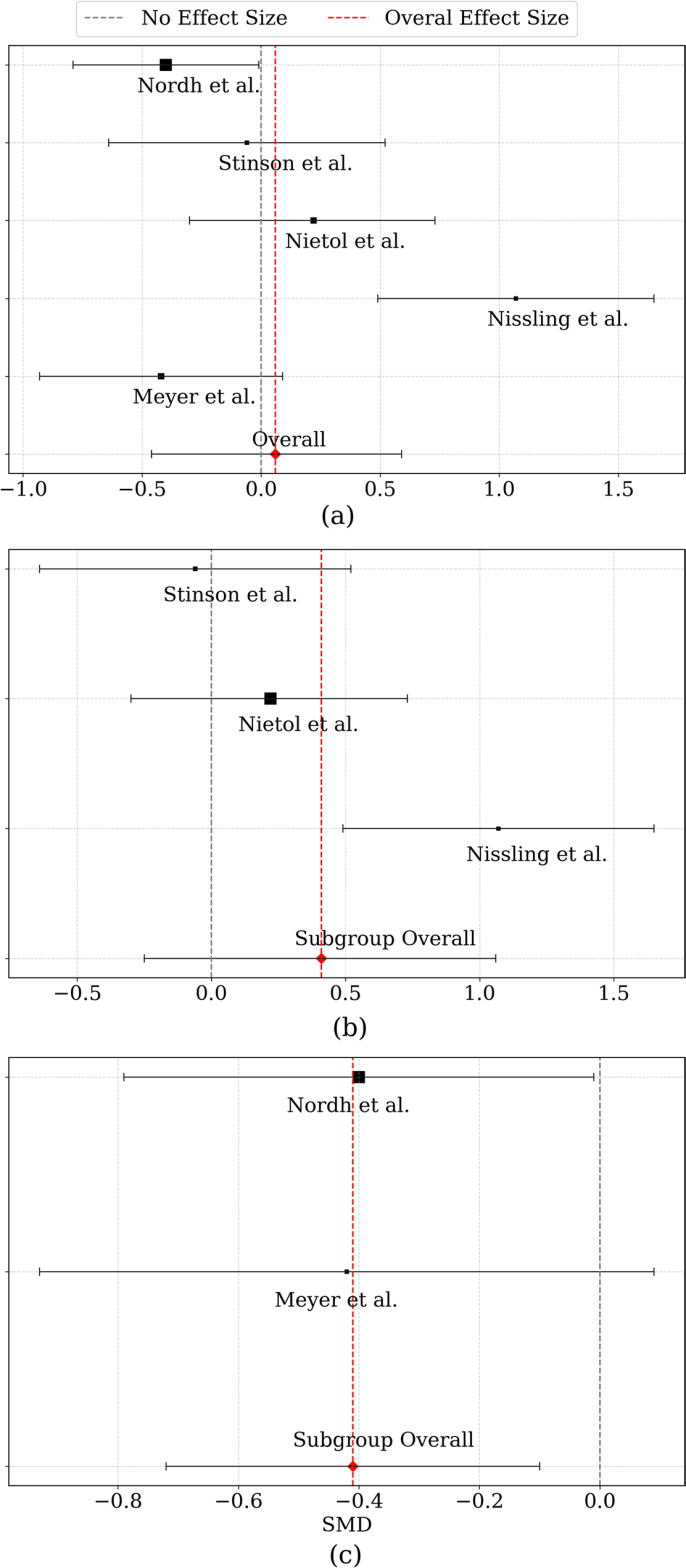
Forest plots illustrating the effects of internet-based interventions on quality of life: **a** overall analysis, **b** subgroup analysis for duration Group A, and **c **subgroup analysis for duration Group B

The summary results of the meta-analysis evaluating the efficacy of internet-based interventions on adolescents’ QoL are presented in Fig. 3 and Table 3. Across all included studies, the overall effect size was small and non-significant (SMD = 0.06, 95% CI: -0.46 to 0.59, *p* = 0.81), with an I² of 81.5%, indicating substantial heterogeneity. This near-zero effect size from a clinical perspective indicates that internet-based interventions, upon summing up the evidence from all included studies, did not really make any significant difference to the quality of life of adolescents. This suggests that simply providing internet-based access to therapeutic content is insufficient to produce tangible improvements in daily functioning, well-being, or life satisfaction across diverse adolescent populations. The forest plot in Fig. 3 illustrates individual study effect sizes and confidence intervals. Some studies, such as Internet-delivered acceptance and commitment therapy [29], reported a significant positive effect (SMD = 1.07, 95% CI: 0.49 to 1.65, *p* < 0.001), whereas others, like Internet-delivered cognitive behavioral therapy [26], showed a small but statistically significant negative effect (SMD = -0.4, 95% CI: -0.79 to -0.01, *p* = 0.04). Remaining studies demonstrated non-significant effects with confidence intervals crossing zero ([27]: SMD = -0.06; [28]: SMD = 0.22; [30]: SMD = -0.42). Study weights were relatively balanced, ranging from 19% to 21.78%, ensuring an equitable contribution to the overall estimate. A supplementary scatter plot in Fig. S2 explored the relationship between participants’ mean age and effect size. The analysis suggests a possible positive correlation, with studies including older adolescents showing stronger positive effects whereas studies with younger participants showed smaller or negligible effects.

**Table 3:** Results of the overall and subgroup analyses of studies on internet-based interventions and their effects on quality of life

Study	SMD	Std. Error	Lower		Upper	*p*-value		Weight	Weight%	Mean Age
[26]	-0.4	0.2	-0.79		-0.01	0.04		3.02	21.78	14.1
[27]	-0.06	0.3	-0.64		0.52	0.84		2.64	19.05	14.6
[28]	0.22	0.26	-0.3		0.73	0.41		2.78	20.05	11.11
[29]	1.07	0.3	0.49		1.65	0		2.63	19	16.63
[30]	-0.42	0.26	-0.93		0.09	0.11		2.79	20.12	13
Overall	0.06	0.27	-0.46		0.59	0.81				
“Heterogeneity: Tau-squared = 0.292, H-squared = 5.412, I-squared = 81.5										
Group A										
[27]	-0.06	0.3	-0.64		0.52	0.84		2.64	19.05	14.6
[28]	0.22	0.26	-0.3		0.73	0.41		2.78	20.05	11.11
[29]	1.07	0.3	0.49		1.65	0		2.63	19	16.63
Subgroup Overall	0.41	0.33	-0.25		1.06	0.23				
Heterogeneity: Tau-squared = 0.254, H-squared = 4.154, I-squared (%) = 75.9										
Group B										
[26]	-0.4	0.2	-0.79	-0.01			0.04	3.02	21.78	14.1
[30]	-0.42	0.26	-0.93	0.09			0.11	2.79	20.12	13
Subgroup Overall	-0.41	0.16	-0.72	-0.1			0.01			

Heterogeneity: Tau-squared = 0.000, H-squared = 1.000, I-squared (%) = 0

Three studies contributed to Duration Group A subgroup illustrated in Fig. 3 (b). The overall effect was positive but non-significant (SMD = 0.41, 95% CI: -0.25 to 1.06, *p* = 0.23), reflecting variability across studies. The largest positive effect was reported by Internet-delivered acceptance and commitment therapy [29] (SMD = 1.07), while [27] showed a negligible effect (SMD = -0.06) and Web-Based Psychosocial Intervention [29] a slight, non-significant positive effect (SMD = 0.22). The trend suggests that older adolescents may benefit more from internet-based interventions, though confidence intervals were wide, indicating variability. Duration Group B subgroup illustrated in Fig. 3 (c) exhibited a small-to-moderate negative overall effect that was statistically significant (SMD = -0.41, 95% CI: -0.72 to -0.10, *p* = 0.01), suggesting that may be associated with lower QoL scores in this limited sample. Individually, Internet-delivered cognitive behavioral therapy [26] showed a significant negative effect (SMD = -0.4, *p* = 0.04), while E-Health Exercise Intervention [30] reported a non-significant negative effect (SMD = -0.42, 95% CI: -0.93 to 0.09). Participants in this subgroup were younger, indicating that early-to-mid adolescents may derive less benefit or even experience negative outcomes from internet-based interventions of this length. The research shows that brief focused digital programs provide small real-world advantages which help people improve their daily emotional state and social interactions and their body health perceptions. However, the lack of statistical significance means that this study cannot confidently recommend these interventions as standalone solutions. Conversely, the negative effect observed in longer interventions raises important clinical concerns as extended digital programs may contribute to intervention fatigue, reduced engagement, or displacement of other beneficial activities, potentially diminishing rather than enhancing quality of life.

### Publication bias

Publication bias refers to the tendency for studies with statistically significant or positive findings to be published more frequently than those with null or negative results. This is a well-recognized issue in meta-analytic research, as it can distort the overall findings and lead to overestimated effect sizes. A common method for detecting publication bias is the funnel plot, which graphs effect sizes (SMD) on the x-axis and standard error (SE) on the y-axis. In the absence of bias, the pattern should resemble an inverted funnel, where larger studies (with lower SE and greater precision) cluster near the top, while smaller studies (with higher SE and lower precision) are symmetrically spread out toward the bottom around the mean effect size. However, asymmetry in the funnel plot suggests possible publication bias, as missing studies typically smaller ones with nonsignificant results create gaps in the distribution. The funnel plot analysis in this study includes research from [26, 30], and [29], with effect sizes ranging from − 1.5 to 2.0 (SMD) and corresponding standard errors between 0.00 and 0.30. Studies with larger SE values (0.20–0.30) are dispersed toward the lower part of the plot, whereas those with smaller SE values (0.00–0.15) cluster near the top. Interestingly, studies with higher SE tend to report positive effect sizes (e.g., 0.5–2.0), while those with lower SE often report negative or null effects (-1.5–0.0), particularly at the lower-precision end. This pattern raises concerns about potential selective reporting. Additionally, the repeated inclusion of [29] in multiple categories warrants further examination to rule out potential dataset duplication. While no direct evidence of duplication was found, verifying the independence of datasets is essential to prevent artificial inflation of effect sizes. Supplementary Fig. S3 presents a funnel plot evaluating potential publication bias across studies investigating the effectiveness of internet-based interventions on adolescent quality of life. The overall plot illustrates the distribution of effect sizes against standard error, where asymmetry may indicate publication bias. Subgroup analyses by Duration A and Duration B examine whether intervention duration influences publication patterns. To complement visual inspection, Egger’s regression test was performed to statistically assess potential publication bias. The test yielded an intercept of -2.424 (SE = 1.712, 95% CI: -7.871 to 3.024, t = -1.416, *p* = 0.252), indicating no significant asymmetry. Given the small number of studies, formal tests of publication bias are underpowered and should be interpreted cautiously.

## Discussion

### Main findings

This meta-analysis examined the impact of internet-based interventions on adolescents’ quality of life by synthesizing findings from multiple trials. The overall SMD was 0.06 (*p* = 0.81), indicating no statistically significant effect. Internet-based interventions did not demonstrate a consistent overall benefit for adolescents’ quality of life across included trials. However, given the limited number of studies and the substantial heterogeneity observed, these findings should be interpreted with caution. The current evidence base remains preliminary, and definitive conclusions regarding effectiveness cannot yet be established. While this suggests that, on average, the interventions had little to no effect on QoL, subgroup analyses provided important insights into potential differential effects based on intervention duration. Subgroup analysis revealed contrasting effects based on intervention length. For brief interventions with shorter duration, the pooled effect size was 0.41 (95% CI: -0.25 to 1.06), suggesting a modest to moderate improvement in QoL, though this result was not statistically significant (*p* = 0.23). Among individual studies in this subgroup [29], reported the most substantial positive effect (SMD = 1.07, *p* < 0.001), whereas other studies showed little to no impact. In contrast, longer-duration interventions yielded a negative pooled effect (SMD = − 0.41, *p* = 0.01). However, this finding should be interpreted with caution, as it was largely driven by a very small number of studies. Therefore, these results do not allow firm conclusions regarding adverse effects of longer interventions. This pattern may reflect differences in adherence demands or engagement over time; however, the limited number of studies precludes reliable conclusions regarding optimal intervention length or comparative effectiveness between intervention formats. Several mechanisms may explain the contrasting effects between brief and longer interventions. Adolescents likely maintain higher adherence and engagement with shorter programs, whereas extended interventions may experience progressive attrition and declining participation, potentially leading to incomplete therapeutic exposure or feelings of failure. Cognitive burden also accumulates differently as brief interventions provide focused content within a manageable timeframe, while prolonged programs may compete with academic demands and developmental tasks, becoming overwhelming rather than supportive. Motivation sustainability is developmentally limited in adolescents; initial enthusiasm may wane over months as circumstances and priorities shift rapidly. Additionally, extended interventions may incur greater opportunity costs by displacing activities that directly enhance quality of life, such as physical exercise, peer interaction, and unstructured leisure. Finally, longer self-guided programs may lack adequate support during motivational dips, whereas brief interventions capitalize on initial engagement. These mechanisms likely interact, suggesting that current delivery models for extended internet-based interventions may not adequately address adolescents’ developmental needs and competing life demands.

Age was explored as a potential moderator of treatment outcomes; however, the available evidence was limited. Studies with younger samples, such as [28], were more likely to obtain neutral or slightly positive effects, while research on older adolescents, such as [29], obtained larger gains. Meta regression analysis explored the correlation between mean age and effect size, yielding a modest positive correlation, though broad confidence intervals moderate confidence in this finding. While adolescence encompasses distinct developmental phases, the limited number of studies and wide confidence intervals prevent firm conclusions regarding differential effects across age groups. Accordingly, any interpretation of age-related differences should be considered exploratory rather than confirmatory.

Beyond statistical outcomes, these findings have important practical implications. The absence of an overall benefit suggests that adolescents receiving internet-based interventions are, on average, experiencing similar quality of life to those receiving usual care. For families and clinicians, this means that while these programs may reduce specific symptoms, they may not translate into broader improvements in how adolescents feel about their lives, engage with peers, perform academically, or participate in meaningful activities. The positive trend in brief interventions suggests that time-limited, focused digital programs may offer the best balance between engagement and benefit, whereas extended programs risk becoming burdensome without proportional gains in overall well-being. Significant heterogeneity was observed among studies (I² = 81.5%), likely stemming from differences in study design, intervention type, and outcome measurement tools. This variability suggests that no single type of internet-based intervention is universally effective. The broad scope of this meta-analysis, which examined various mental health conditions among adolescent student populations, contributed to this heterogeneity. While subgroup analyses were conducted to address this issue, unexplained variability may impact the robustness of the conclusions, and results should be interpreted with caution.

### Study limitations

Several limitations warrant consideration. The primary limitation is the relatively small number of RCTs included, which restricted statistical power and made it difficult to formally assess for publication bias. Because only five studies were available, this study could not reliably test for small-study or publication bias; thus, the possibility of bias cannot be ruled out. To address this, this study clearly documented the search strategy, inclusion and exclusion criteria, study selection process, and statistical analysis plan, thereby providing a verifiable account of the procedures followed. The long-term effectiveness of internet-based interventions remains uncertain, as it is unclear whether observed benefits persist beyond the intervention period. The exclusion of children with intellectual and developmental disabilities from several studies limits generalizability, despite the high prevalence of mental health disorders within this population. Additionally, while some studies achieved statistically significant symptom reduction, whether these changes translate into practical functional improvements remains unknown. Generalizability is further limited because all included RCTs were conducted in high-income countries. The availability and effectiveness of digital interventions may vary depending on economic and cultural contexts. Socioeconomic determinants, information infrastructure, and cultural attitudes toward mental health interventions may influence the degree to which adolescents utilize these treatments in low- and middle-income settings.

### Future research directions

Future research should prioritize several key areas. Studies with extended follow-up periods are needed to assess the durability of intervention effects and determine whether benefits persist or diminish over time. Research should stratify results by developmental stage to provide deeper insights into how cognitive and emotional maturation affects treatment outcomes. Comparative studies must assess the impact of guided versus unguided internet-delivered interventions and contrast their effectiveness with traditional face-to-face treatments. Understanding optimal treatment duration, delivery modes, and specific therapeutic techniques will be crucial for identifying the most efficacious approaches for different adolescent populations. Research must also examine the availability and efficacy of web-based interventions among marginalized groups, including adolescents in low- and middle-income nations and individuals with intellectual or developmental disabilities. Determining how interventions can be adapted to benefit these underrepresented populations will ensure that internet-based treatments are equitable and effective across diverse contexts. Future meta-analyses should examine whether intervention effects align with clinically established thresholds to determine their practical applicability and whether changes translate into meaningful functional improvements.

### Clinical implications

This meta-analysis found no significant overall effect of internet-based interventions on adolescents’ QoL, with notable heterogeneity across studies. Brief interventions showed a trend toward modest benefits, whereas findings for longer interventions were inconsistent and based on limited evidence, underscoring the need for cautious interpretation. Age and developmental stage appear to influence treatment response, with suggestions that older adolescents may benefit more than younger ones. For clinical practice, internet-based interventions should not be considered uniformly beneficial for all adolescents. Clinicians may consider prioritizing brief, structured internet-based interventions to enhance engagement and reduce intervention fatigue, as shorter programs demonstrated more consistent trends toward benefit. Intervention duration should therefore be selected deliberately rather than assumed to be neutral. Monitoring adolescents’ digital workload and overall screen exposure during treatment is advisable to prevent unintended burden or disengagement. Regular progress evaluation and proactive follow-up, particularly in longer programs, are recommended to sustain motivation and identify early signs of reduced adherence. These measures are especially relevant given the variability in outcomes observed across studies. Age-appropriate tailoring remains essential, and interventions should be aligned with the adolescent’s developmental stage and specific psychosocial context. Integrating digital interventions with structured clinician guidance may further optimize outcomes compared with unguided self-help approaches. In addition, combining internet-based interventions with conventional healthcare services or adjunctive treatments, when clinically indicated, may contribute to more comprehensive and coordinated care. As the evidence base continues to evolve, clinicians should remain attentive to emerging data clarifying which subgroups of adolescents and which clinical conditions derive the greatest benefit from digital modalities, ensuring that implementation strategies remain evidence-informed and equitable.

## Supplementary Information

Supplementary Material 1.

Supplementary Material 2.

Supplementary Material 3.

Supplementary Material 4.

Supplementary Material 5.

Supplementary Material 6.

Supplementary Material 7.

## Data Availability

The datasets used and/or analyzed during the current study are available from the corresponding author on reasonable request.
