# Supplementary material for: The role of internet-based therapies in adolescents’ quality of life: a systematic review and meta-analysis
Source: BMC Psychol. 2026 Mar 14;14:587. doi: 10.1186/s40359-026-04285-z (PMC13101184; doi:10.1186/s40359-026-04285-z)
Supplement: Supplementary file 2 — Supplementary Material 2. [file 40359_2026_4285_MOESM2_ESM.pdf]

Meta-Analysis: Continuous Outcomes with Raw Data

Notes

Output Created		18-FEB-2025 13:00:18
Comments		
Input	Active Dataset	DataSet1
	Filter	<none>
	Weight	<none>
	Split File	<none>
	N of Rows in Working Data File	25
Missing Value Handling	Definition of Missing	User-defined missing values are treated as missing.
	Cases Used	Each statistic is based on all valid data for the available variable(s) used in computing the statistic.

Notes

Syntax	META CONTINUOUS<br>/DATA TREATMENT=N (Interventionsamplesize) MEAN (InterventionMeanChange) STD (InterventionSDChange)<br>CONTROL=N (Controlsamplesize) MEAN (ControlMeanChange) STD(ControlSDChange) ID=Author<br>ESTYPE=COHEN_D<br>/CRITERIA CILEVEL=95 SCOPE=AVAILABLE CLASSMISSING=EXCLUDE MAXITER=100 MAXSTEP=5<br>CONVERGENCE=0.000001<br>/INFERENCE MODEL=RANDOM ESTIMATE=REML ADJUSTSE=NONE<br>/BIAS INTERCEPT=INCLUDE DISTRIBUTION=T<br>/PRINT HOMOGENEITY HETEROGENEITY<br>/FORESTPLOT DISPLAY=ES SE CI PVAL WEIGHT POSITION=RIGHT ANNOTATIONS=HOMOGENEITY HETEROGENEITY<br>REFLINES=OVERALL NULL<br>/BUBBLEPLOT PROPORTION=TRUE FITLINE=TRUE CI=TRUE PREDICTORS=Lengthofinterventionweek<br>CENTER=FALSE LABEL=Author(AUTO)<br>/FUNNELPLOT YAXIS=SE LABEL=Author (AUTO)<br>/GALBRAITHPLOT CI=TRUE LABEL=Author (AUTO).

Notes

Resources	Processor Time	00:00:01.58
	Elapsed Time	00:00:01.09

[DataSet1]

Meta-Analysis Summary

Data Type	Raw
Outcome Type	Continuous
Effect Size Measure	Cohen's d
Model	Random-effects
Weight	Inverse-variance[a]
Estimation Method	REML
Standard Error Adjustment	None

a. Random-effects weights including both within- and between-study variance.

Case Processing Summary

	N	Percent
Included	5	20.0%
Missing	20	80.0%
Invalid[a]	0	0.0%
Total	25	100.0%

a. Nonpositive variance or standard error, or insufficient study size.

Effect Size Estimates

	Effect Size	Std. Error	Z	Sig. (2-tailed)	95% Confidence Interval	
					Lower	Upper
Overall	.065	.2687	.240	.810	-.462	.591

Test of Homogeneity

	Chi-square (Q statistic)	df	Sig.
Overall	20.363	4	<.001

Heterogeneity Measures

Overall	Tau-squared	.292
	H-squared	5.412
	I-squared (%)	81.5

Egger's Regression-Based Test[a]

Parameter	Coefficient	Std. Error	t	Sig. (2-tailed)	95% Confidence Interval	
					Lower	Upper
(Intercept)	-2.424	1.7117	-1.416	.252	-7.871	3.024
SE[b]	9.555	6.5236	1.465	.239	-11.206	30.316

a. Random-effects meta-regression

b. Standard error of effect size

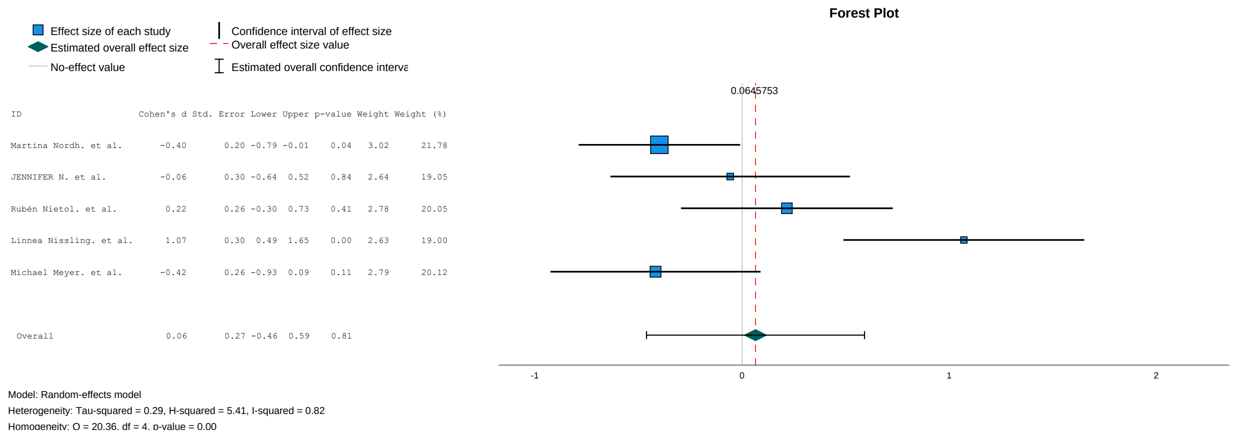

Bubble Plot

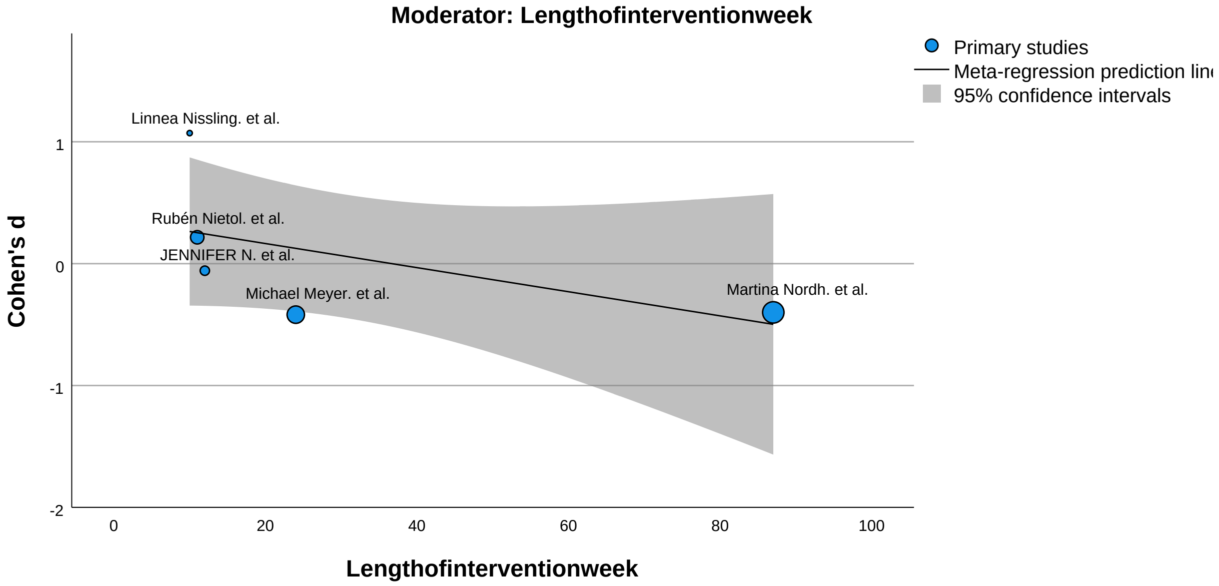

Model: Random-effects model

Weights: Random-effects

Funnel Plot

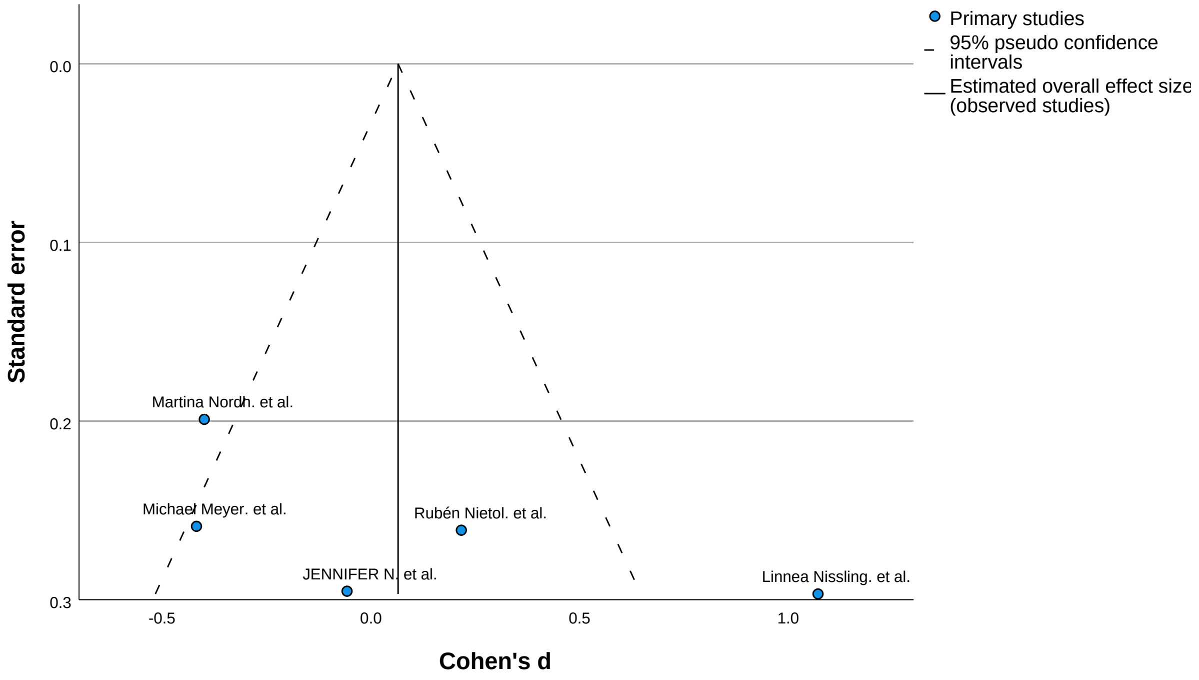

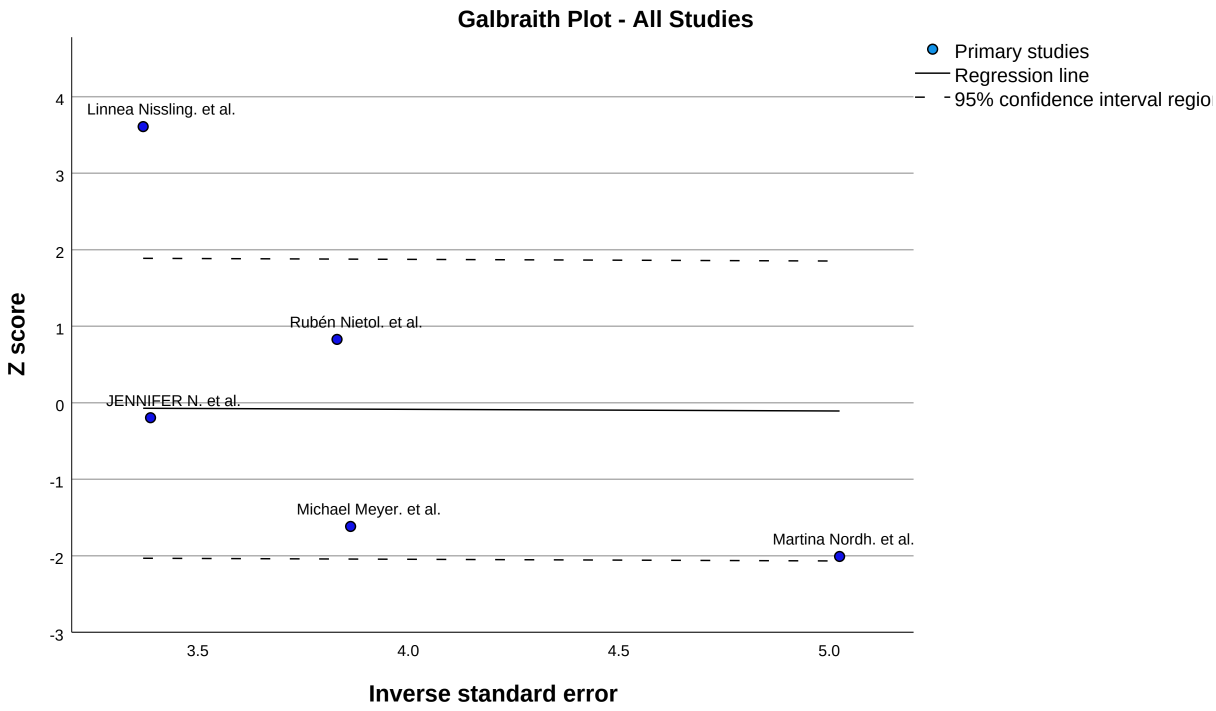

Galbraith Plot - All Studies
Primary studies
Regression line
95% confidence interval regio
Z score
4
3
2
1
0
-1
-2
-3
Linnea Nissling. et al.
Rubén Nietol. et al.
JENNIFER N. et al.
Michael Meyer. et al.
Martina Nordh. et al.
3.5
4.0
4.5
5.0
Inverse standard error
