# Supplementary material for: The role of internet-based therapies in adolescents’ quality of life: a systematic review and meta-analysis
Source: BMC Psychol. 2026 Mar 14;14:587. doi: 10.1186/s40359-026-04285-z (PMC13101184; doi:10.1186/s40359-026-04285-z)
Supplement: Supplementary file 3 — Supplementary Material 3. [file 40359_2026_4285_MOESM3_ESM.docx]

**Identification of studies via databases and registers**

Records removed *before screening*:

Duplicate records removed

(n = 83)

Records identified through database searching

PubMed (n = 38)

Scopus (n = 228)

WOS (n= 72)

**Identification**

Records excluded:

Excluded based on inclusion and exclusion criteria (n = 210)

Remaining records for title and abstract screening (n = 255)

**Screening**

Records excluded:

Not extractable data for synthesis (n = 40)

Remaining records for full-text screening (n= 45)

Studies included in meta-analysis (n = 5)

**Included**

*Consider, if feasible to do so, reporting the number of records identified from each database or register searched (rather than the total number across all databases/registers).

**If automation tools were used, indicate how many records were excluded by a human and how many were excluded by automation tools.

Source: Page MJ, et al. BMJ 2021;372:n71. doi: 10.1136/bmj.n71.

This work is licensed under CC BY 4.0. To view a copy of this license, visit <https://creativecommons.org/licenses/by/4.0/>
