# Supplementary material for: The role of internet-based therapies in adolescents’ quality of life: a systematic review and meta-analysis
Source: BMC Psychol. 2026 Mar 14;14:587. doi: 10.1186/s40359-026-04285-z (PMC13101184; doi:10.1186/s40359-026-04285-z)
Supplement: Supplementary file 4 — Supplementary Material 4. [file 40359_2026_4285_MOESM4_ESM.pdf]

Meta-Analysis: Continuous Outcomes with Raw Data

Notes

Output Created		18-FEB-2025 13:32:52
Comments		
Input	Active Dataset	DataSet1
	Filter	<none>
	Weight	<none>
	Split File	<none>
	N of Rows in Working Data File	25
Missing Value Handling	Definition of Missing	User-defined missing values are treated as missing.
	Cases Used	Each statistic is based on all valid data for the available variable(s) used in computing the statistic.

Notes

Syntax	META CONTINUOUS /DATA TREATMENT=N (Interventionsamplesize) MEAN (InterventionMeanChange) STD (InterventionSDChange) CONTROL=N (Controlsamplesize) MEAN (ControlMeanChange) STD(ControlSDChange) ID=Author ESTYPE=COHEN_D /CRITERIA CILEVEL=95 SCOPE=AVAILABLE CLASSMISSING=EXCLUDE MAXITER=100 MAXSTEP=5 CONVERGENCE=0.000001 /ANALYSIS SUBGROUP=Lengthofinterventionweek /INFERENCE MODEL=RANDOM ESTIMATE=REML ADJUSTSE=NONE /BIAS INTERCEPT=INCLUDE DISTRIBUTION=T /PRINT HOMOGENEITY HETEROGENEITY /FORESTPLOT DISPLAY=ES SE CI PVAL WEIGHT POSITION=RIGHT ANNOTATIONS=HOMOGENEITY HETEROGENEITY REFLINES=OVERALL NULL /BUBBLEPLOT PROPORTION=TRUE FITLINE=TRUE CI=TRUE PREDICTORS=MeanAge CENTER=FALSE LABEL=Author(AUTO) /FUNNELPLOT YAXIS=SE LABEL=Author (AUTO) /GALBRAITHPLOT CI=TRUE LABEL=Author (AUTO).

Notes

Resources	Processor Time	00:00:03.56
	Elapsed Time	00:00:01.48

```
[DataSet1]
```

Meta-Analysis Summary

Data Type	Raw
Outcome Type	Continuous
Effect Size Measure	Cohen's d
Model	Random-effects
Weight	Inverse-variance[a]
Estimation Method	REML
Standard Error Adjustment	None
Subgroup Analysis	Length of intervention (week)

a. Random-effects weights including both within- and between-study variance.

Case Processing Summary

	N	Percent
Included	5	20.0%
Missing	20	80.0%
Invalid[a]	0	0.0%
Total	25	100.0%

a. Nonpositive variance or standard error, or insufficient study size.

Effect Size Estimates for Subgroup Analysis

	Effect Size	Std. Error	Z	Sig. (2-tailed)	95% Confidence Interval	
					Lower	Upper
a	.405	.3342	1.213	.225	-.250	1.061
b	-.407	.1578	-2.578	.010	-.716	-.098
Overall	.065	.2687	.240	.810	-.462	.591

Test of Homogeneity

	Chi-square (Q statistic)	df	Sig.
a	8.010	2	.018
b	.003	1	.954
Overall	20.363	4	<.001

Test of Subgroup Homogeneity

	Chi-square (Q statistic)	df	Sig.
Length of intervention (week)	4.830	1	.028

Heterogeneity Measures

a	Tau-squared	.254
	H-squared	4.154
	I-squared (%)	75.9
b	Tau-squared	.000
	H-squared	1.000
	I-squared (%)	.0
Overall	Tau-squared	.292
	H-squared	5.412
	I-squared (%)	81.5

Egger's Regression-Based Test[a,b]

	Parameter	Coefficient	Std. Error	t	Sig. (2-tailed)	95% Confidence Interval	
						Lower	Upper
a	(Intercept)	-2.245	7.7841	-.288	.821	-101.151	96.660
	SE[c]	9.338	27.3521	.341	.791	-338.203	356.879
Overall	(Intercept)	-2.424	1.7117	-1.416	.252	-7.871	3.024
	SE[c]	9.555	6.5236	1.465	.239	-11.206	30.316

a. Random-effects meta-regression

b. Regression Based Test cannot be computed for subgroup(s) Length of intervention(week) = b.

c. Standard error of effect size

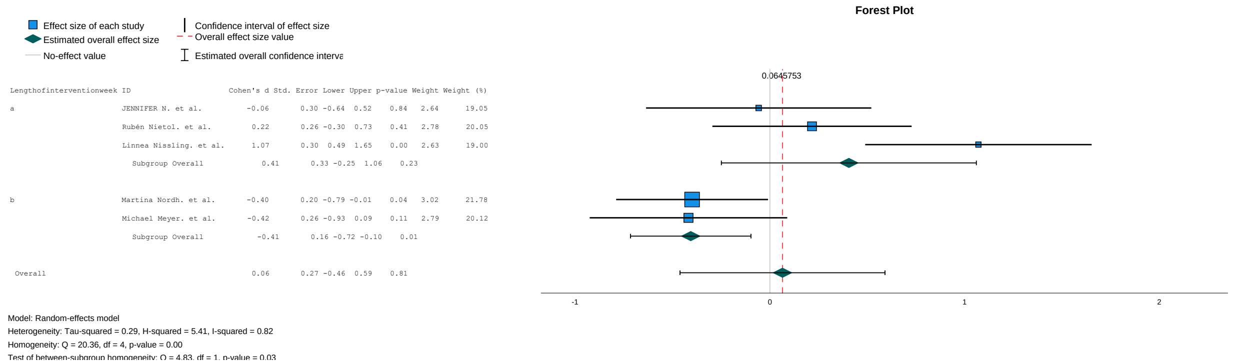

Lengthofinterventionweek	ID	Cohen's d	Std. Error	Lower	Upper	p-value	Weight	Weight (%)
a	JENNIFER N. et al.	-0.06	0.30	-0.64	0.52	0.84	2.64	19.05
	Rubén Nietol. et al.	0.22	0.26	-0.30	0.73	0.41	2.78	20.05
	Linnea Nissling. et al.	1.07	0.30	0.49	1.65	0.00	2.63	19.00
	Subgroup Overall	0.41	0.33	-0.25	1.06	0.23		
b	Martina Nordh. et al.	-0.40	0.20	-0.79	-0.01	0.04	3.02	21.78
	Michael Meyer. et al.	-0.42	0.26	-0.93	0.09	0.11	2.79	20.12
	Subgroup Overall	-0.41	0.16	-0.72	-0.10	0.01		
Overall		0.06	0.27	-0.46	0.59	0.81		

Model: Random-effects model
Heterogeneity: Tau-squared = 0.29, H-squared = 5.41, I-squared = 0.82
Homogeneity: Q = 20.36, df = 4, p-value = 0.00
Test of between-subgroup homogeneity: Q = 4.83, df = 1, p-value = 0.03

Bubble Plot - All Studies

Moderator: MeanAge

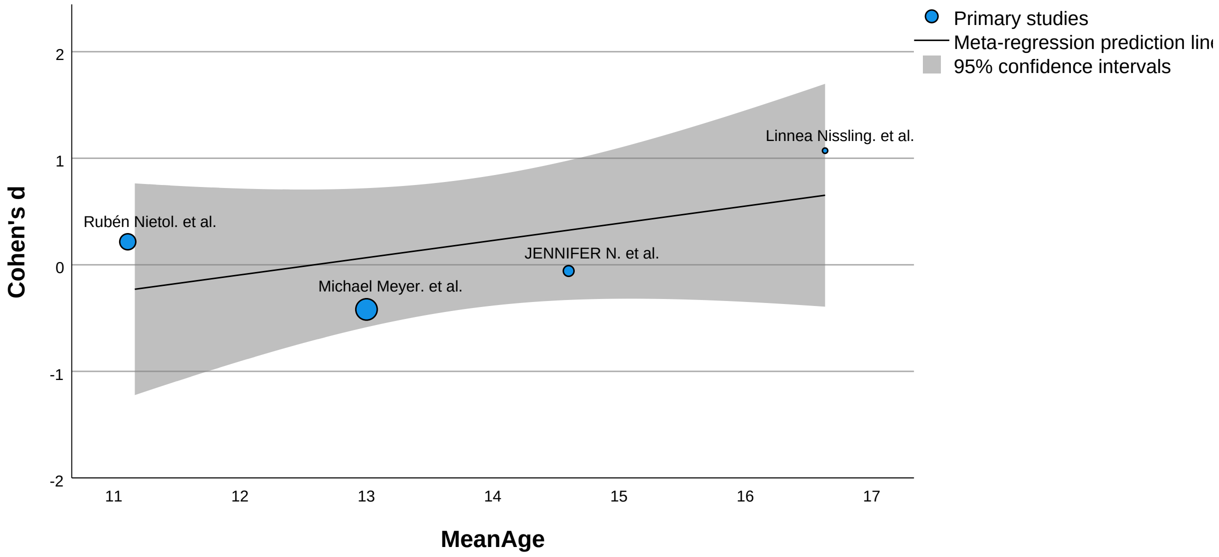

Model: Random-effects model

Weights: Random-effects

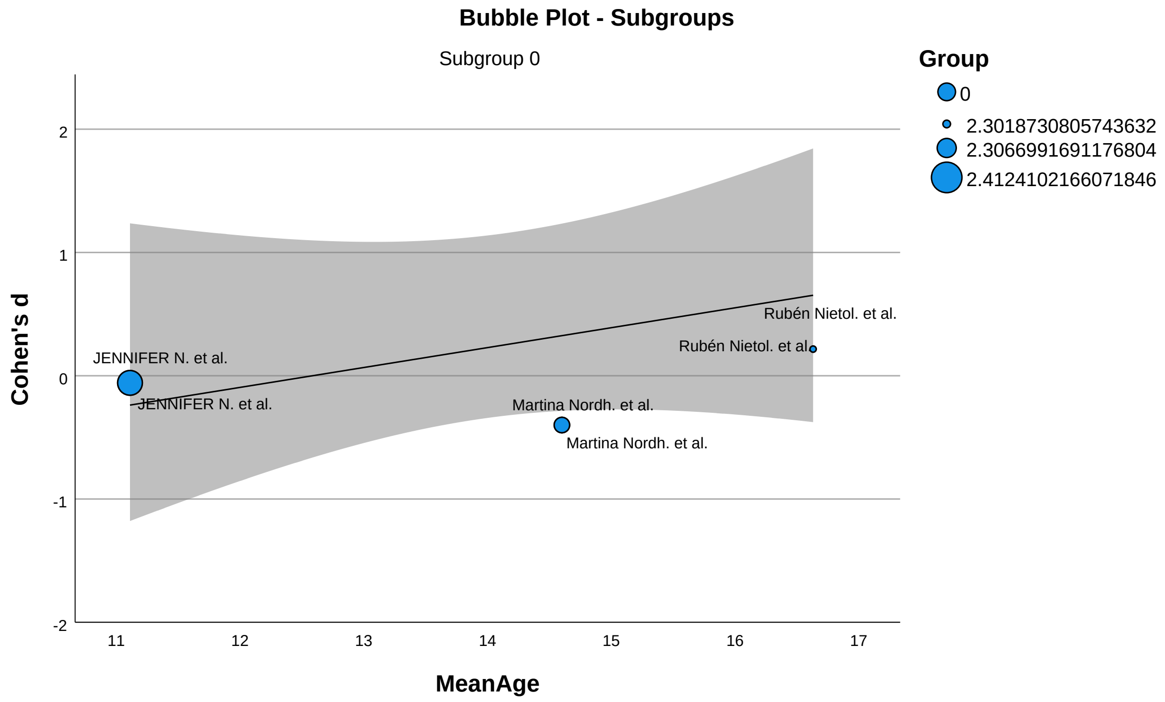

Bubble Plot - Subgroups
Subgroup 0
Group
0
2.3018730805743632
2.3066991691176804
2.4124102166071846
Cohen's d
2
1
0
-1
-2
JENNIFER N. et al.
JENNIFER N. et al.
Rubén Nietol. et al.
Rubén Nietol. et al.
Martina Nordh. et al.
Martina Nordh. et al.
11
12
13
14
15
16
17
MeanAge

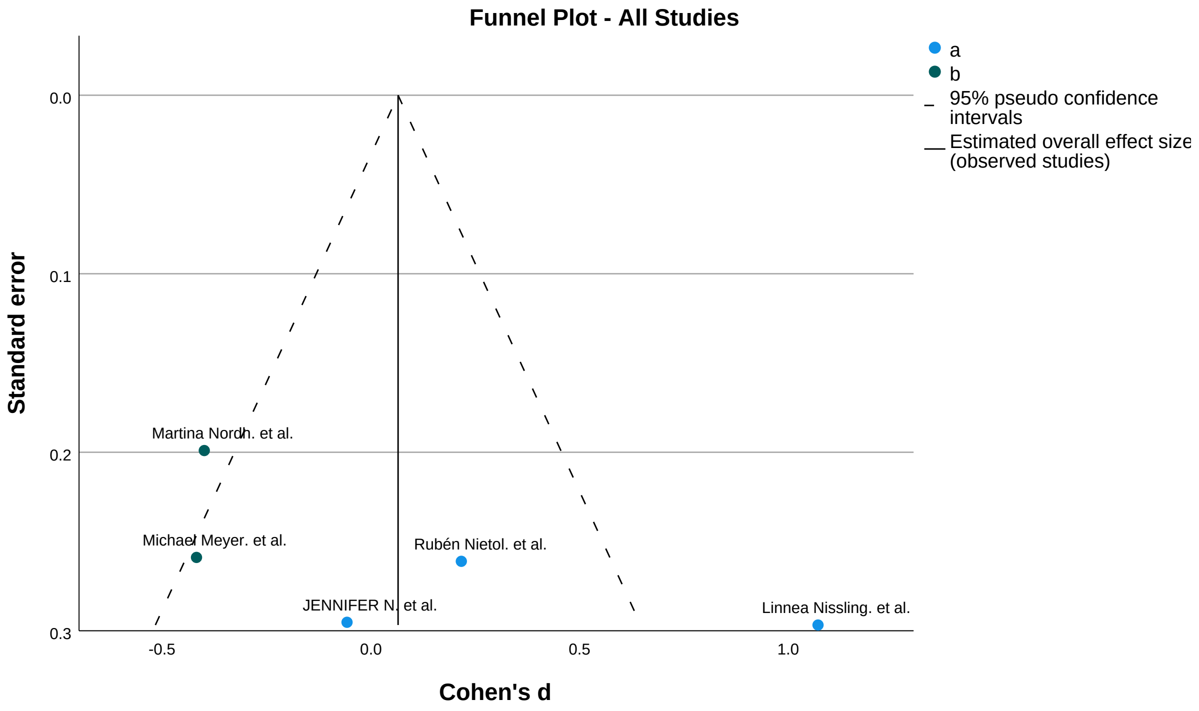

Funnel Plot - All Studies
a
b
95% pseudo confidence intervals
Estimated overall effect size (observed studies)
Standard error
0.0
0.1
0.2
0.3
Martina Nordh. et al.
Michael Meyer. et al.
Rubén Nietol. et al.
JENNIFER N. et al.
Linnea Nissling. et al.
-0.5
0.0
0.5
1.0
Cohen's d

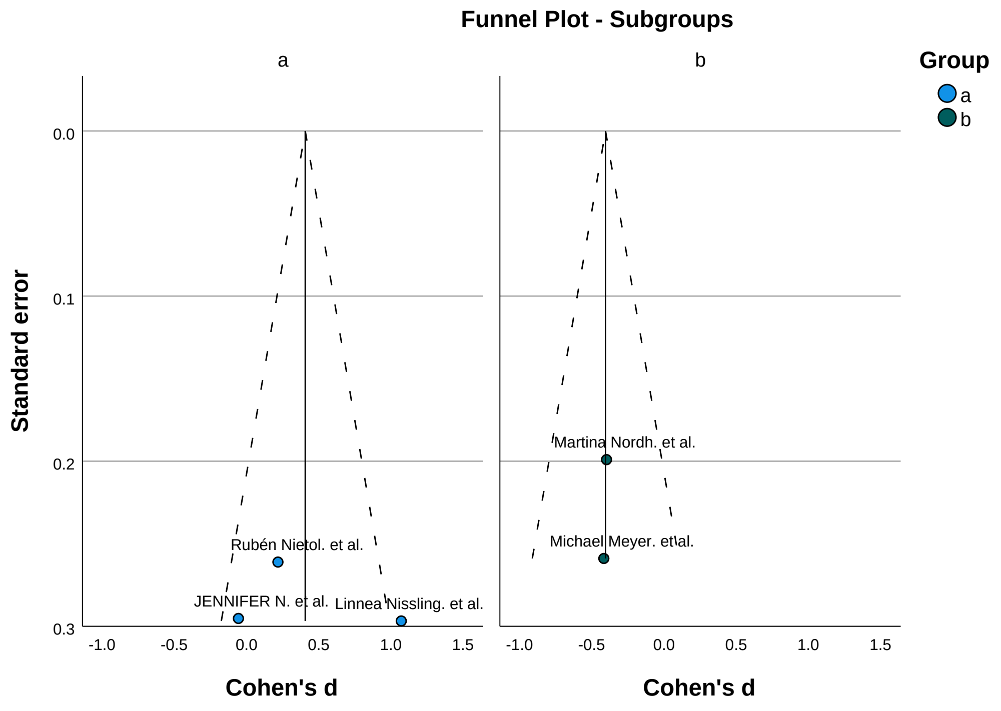
Funnel Plot - Subgroups
a
b
Group
a
b
Standard error
0.0
0.1
0.2
0.3
Martina Nordh. et al.
Michael Meyer. et al.
Rubén Nietol. et al.
JENNIFER N. et al.
Linnea Nissling. et al.
-1.0
-0.5
0.0
0.5
1.0
1.5
Cohen's d
Cohen's d

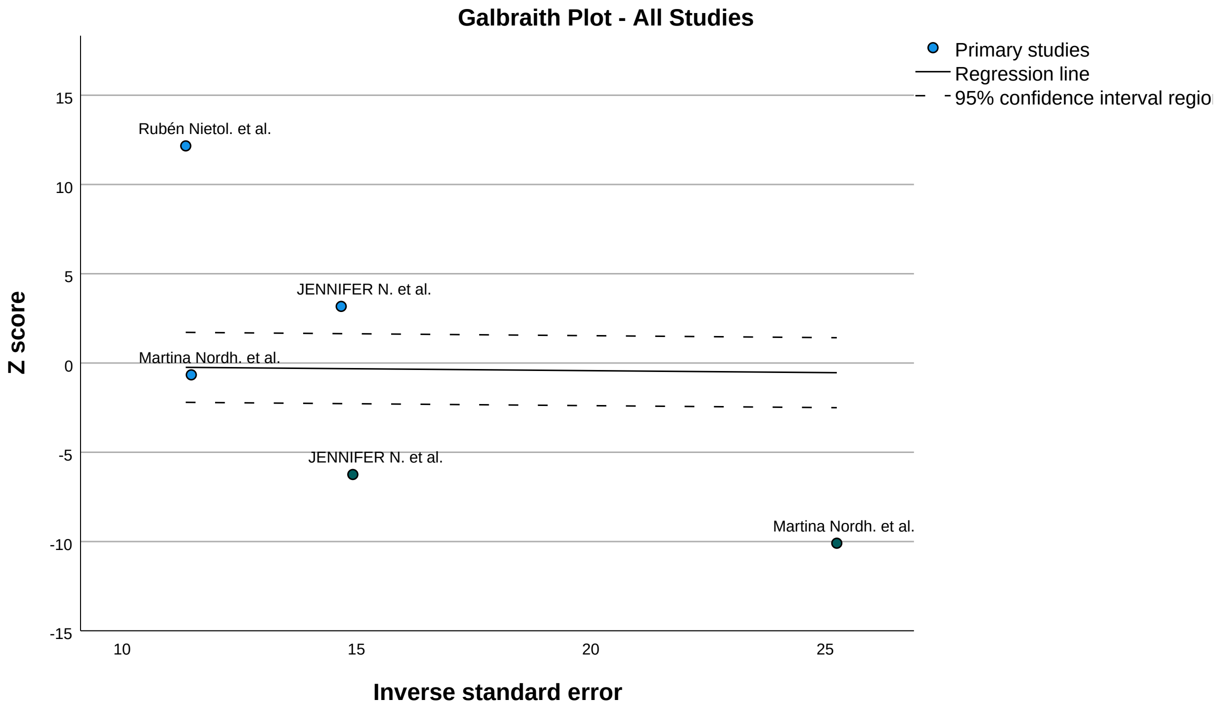
Galbraith Plot - All Studies
Primary studies
Regression line
95% confidence interval regio
Z score
15
10
5
0
-5
-10
-15
Rubén Nietol. et al.
JENNIFER N. et al.
Martina Nordh. et al.
JENNIFER N. et al.
Martina Nordh. et al.
10
15
20
25
Inverse standard error

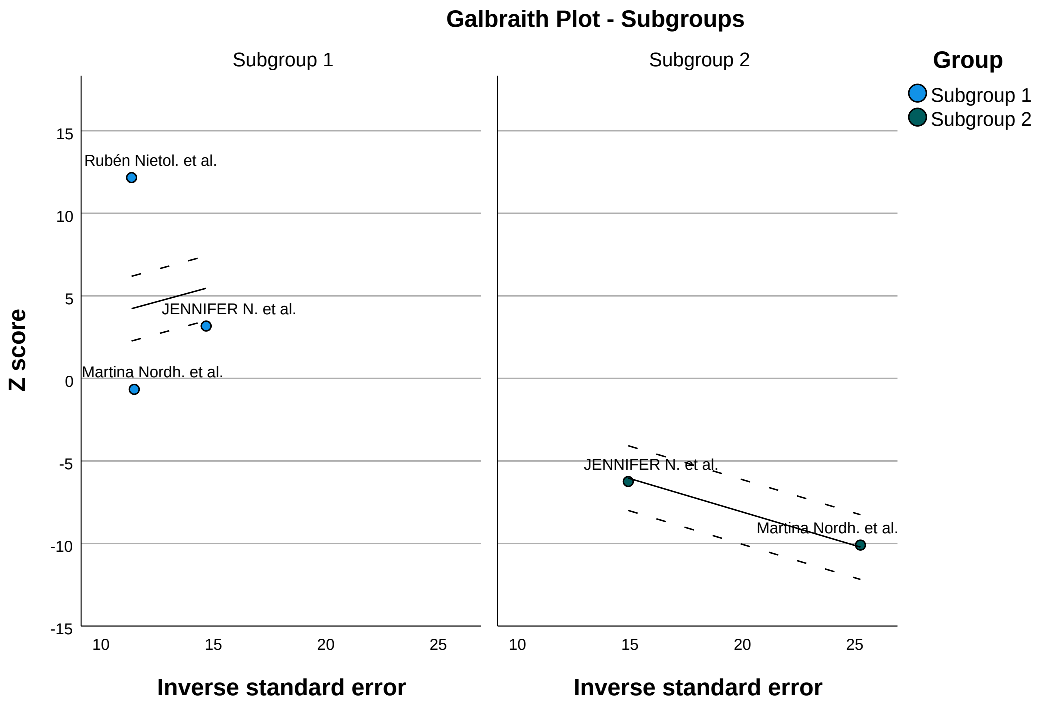

Galbraith Plot - Subgroups
Subgroup 1
Subgroup 2
Group
Subgroup 1
Subgroup 2
Z score
15
10
5
0
-5
-10
-15
Rubén Nietol. et al.
JENNIFER N. et al.
Martina Nordh. et al.
JENNIFER N. et al.
Martina Nordh. et al.
10
15
20
25
10
15
20
25
Inverse standard error
Inverse standard error
